# P-11. Assessing the Epidemiological Impact of an Age-Based Pneumococcal Vaccine Recommendation in France

**DOI:** 10.1093/ofid/ofae631.221

**Published:** 2025-01-29

**Authors:** Kevin Bakker, Rachel Oidtman, Giulio Meleleo, Oluwaseun Sharomi, Manon Breau Brunel, Gaelle Farge, Jeremy Carette, Robert Nachbar

**Affiliations:** Merck & Co., Inc., Rahway, NJ, USA, Philadelphia, Pennsylvania; Merck & Co., Inc., Lansdale, Pennsylvania; Wolfram Research, Inc., Champaign, Illinois; Merck & Co., Inc., Lansdale, Pennsylvania; MSD, Paris, France, Paris, Ile-de-France, France; MSD, Paris, France, Paris, Ile-de-France, France; MSD, Paris, France, Paris, Ile-de-France, France; Wolfram Research, Inc., Champaign, Illinois

## Abstract

**Background:**

In France, pneumococcal vaccination recommendations exist for children aged 0-2 years and for adults with certain risk conditions. Despite most countries having age-based recommendations in adults over the age of 60 or 65 years, there is no adult age-based recommendation in France. Since May 2024, PCV20, a 20-valent pneumococcal conjugate vaccine (PCV) has been recommended in adults with risk conditions. V116 is a new adult focused PCV developed to prevent the 21 serotypes that occur most commonly in adults (86%of IPD).

Table 1:

**Figure:**
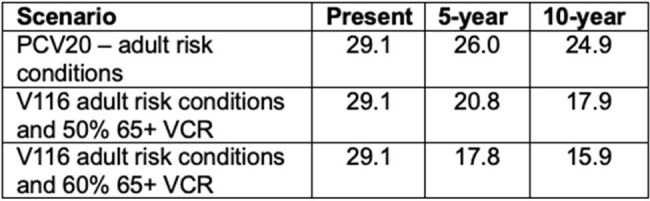

IPD incidence per 100,000 individuals in the 65+ year old population.

**Methods:**

We evaluated the impact of introducing an age-based pneumococcal vaccine recommendation in 65+ year-old individuals in France using a compartmental dynamic transmission model which tracked age- and serotype-specific *Streptococcus pneumoniae* (SP) transmission. The model incorporated the historical introductions of multiple age- and serotype-specific pneumococcal vaccines and accounted for both herd protection from pediatric vaccination and serotype replacement from vaccine introductions while it was calibrated to historical age- and serotype-specific invasive pneumococcal disease (IPD) data spanning 2000-2019. We evaluated the impact of introducing V116 into the at-risk adult population and in individuals aged 65+ years-old. For projections we maintained a 50/50 coverage ratio of PCV13/PCV15 in 95% of children and assumed a vaccine coverage rate (VCR) of 50% or 60% in adults aged 65+ years old. We compared this strategy against the current recommendation of PCV20 in adult risk populations only.

Table 2: Cumulative IPD cases in all ages for different projection periods.

**Figure:**
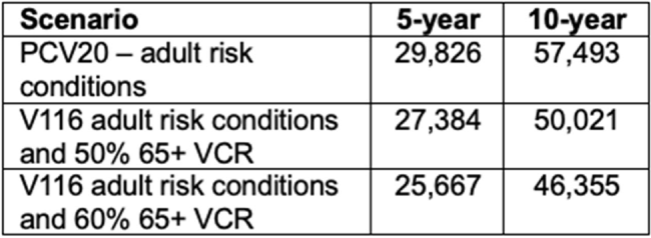

**Results:**

In the first 10 years, the introduction of V116 into an age-based recommendation in France reduced IPD incidence in adults 65+ years old from 29.1 cases/100k to 17.9 or 15.9/100k if VCR of V116 was set to 50% or 60%, respectively. Continued use of PCV20 in adult risk populations led to 24.9 cases/100k (Table 1). This equated to a total IPD case count (all ages) of 57,493 with PCV20, 50,021 with 50% V116 VCR, and 46,355 with 60% V116 VCR (Table 2).

**Conclusion:**

Introducing an age-based pneumococcal vaccine recommendation in France led to substantial reductions in population level IPD. After 10 years, V116 with a 60% VCR led to ∼11,000 fewer cases than the continued use of PCV20 in risk populations.

**Disclosures:**

**Kevin Bakker, PhD**, Merck & Co., Inc.: Grant/Research Support|Merck & Co., Inc.: Stocks/Bonds (Public Company) **Rachel Oidtman, PhD**, Merck & Co., Inc.: Full time employee|Merck & Co., Inc.: Stocks/Bonds (Public Company) **Giulio Meleleo, PhD**, Merck & Co., Inc.: Vendor **Oluwaseun Sharomi, MSc, PhD**, Merck & Co., Inc.: Full time employee|Merck & Co., Inc.: Stocks/Bonds (Public Company) **Manon Breau Brunel, n/a**, MSD: Grant/Research Support|MSD: Stocks/Bonds (Public Company) **Gaelle Farge, n/a**, MSD: Grant/Research Support|MSD: Stocks/Bonds (Public Company) **Jeremy Carette, n/a**, MSD: Advisor/Consultant **Robert Nachbar, PhD**, Merck & Co., Inc.: US 7,219,020, US 5,292,741|Merck & Co., Inc.: Vendor|Merck & Co., Inc.: Stocks/Bonds (Public Company)

